# Supplementary material for: Regulatory-approved deep learning/machine learning-based medical devices in Japan as of 2020: A systematic review
Source: PLOS Digit Health. 2022 Jan 18;1(1):e0000001. doi: 10.1371/journal.pdig.0000001 (PMC9931274; doi:10.1371/journal.pdig.0000001)
Supplement: S1 Table — (DOCX) [file pdig.0000001.s001.docx]

**Supplementary Table.** **Summary of the ML/DL-based medical devices without a response from the marketing authorization holders.**

| No. | Name of device or algorithm | Short description | Class | Name of company | Application distinction | Approval date | PMDA approval number | Medical specialty | Algorithm | CAD | country | Image Type |
| --- | --- | --- | --- | --- | --- | --- | --- | --- | --- | --- | --- | --- |
| 1 | ECG App | Take electrocardiogram / Classify heart rate | Ⅱ | Apple Inc． | approved | 4-Sep-20 | 30200BZI00020000 | cardiology | - | CADe | US | ECG |
| 2 | Irregular Rhythm Notification Feature | Check heart rhythms/ Alert if atrial fibrillation is identified | Ⅱ | Apple Inc. | approved | 4-Sep-20 | 30200BZI00021000 | cardiology | - | CADe | US | Photoplethysmography |
