# Supplementary material for: Regulatory-approved deep learning/machine learning-based medical devices in Japan as of 2020: A systematic review
Source: PLOS Digit Health. 2022 Jan 18;1(1):e0000001. doi: 10.1371/journal.pdig.0000001 (PMC9931274; doi:10.1371/journal.pdig.0000001)
Supplement: S1 Note — Introduction of the national strategy taken in Japan for earlier promotion and approval of software as a medical device. (DOCX) [file pdig.0000001.s002.docx]

**Supplemental Note**

*Digital Transformation Action Strategies in Healthcare for software as a medical device*

The Ministry of Health Labor and Welfare and PMDA are taking a strategic approach to promoting early approval of SaMD. This package is named the Digital Transformation Action Strategies in Healthcare (DASH) for SaMD. This package of strategies incorporates the following strategies: 1) promote the identification of innovative seeds of SaMD in the early phase to clarify the approval criteria in advance, 2) establish a unified consultation desk that provides the appropriate contact according to the contents of the consultation, 3) update the approval procedures to address the particularities of SaMD, and 4) strengthen the organizational structure involved in the review process of SaMD.

In particular, a new approval system called “Improvement Design within Approval for Timely Evaluation and Notice” (IDATEN) has been introduced to simplify the approval process for partial change. “IDATEN” is named after Mahayana bodhisattva Idaten, who is the god of speed in Japan. PMDA’s official announcement (24th May, 2021) explains IDATEN as follows: “In light of the nature of medical devices that undergo ongoing modifications and improvements during their post-market lifecycles, and AI-based programs and software whose performance is constantly changing and improving, change plans will be confirmed during the approval review process so that partial amendments to approvals can be made promptly within the scope of such plans during the devices’ post-market lifecycles.” (https://www.pmda.go.jp/english/about-pmda/0006.pdf, accessed on 3rd, September, 2021).
