# Supplementary material for: Regulatory-approved deep learning/machine learning-based medical devices in Japan as of 2020: A systematic review
Source: PLOS Digit Health. 2022 Jan 18;1(1):e0000001. doi: 10.1371/journal.pdig.0000001 (PMC9931274; doi:10.1371/journal.pdig.0000001)

**Supplementary Figure**

Supplementary Figure 1. Global market share of６major medical devices field (2014)

This figure shows the market share by country in each medical devices field. The Original data are provided by the Japan Ministry of Economy, Trade, and Industry (available at <https://www.med-device.jp/repository/meti-seisaku-202002.pdf>, accessed on 20^th^ August 2021). Because the original data were provided in Japanese, we translated them into English. Japanese Yen was converted to US dollars based on an exchange rate of 2014 ($1≒109.66yen). The figure shows that the Japanese companies dominate the global market share of the endoscopic market.


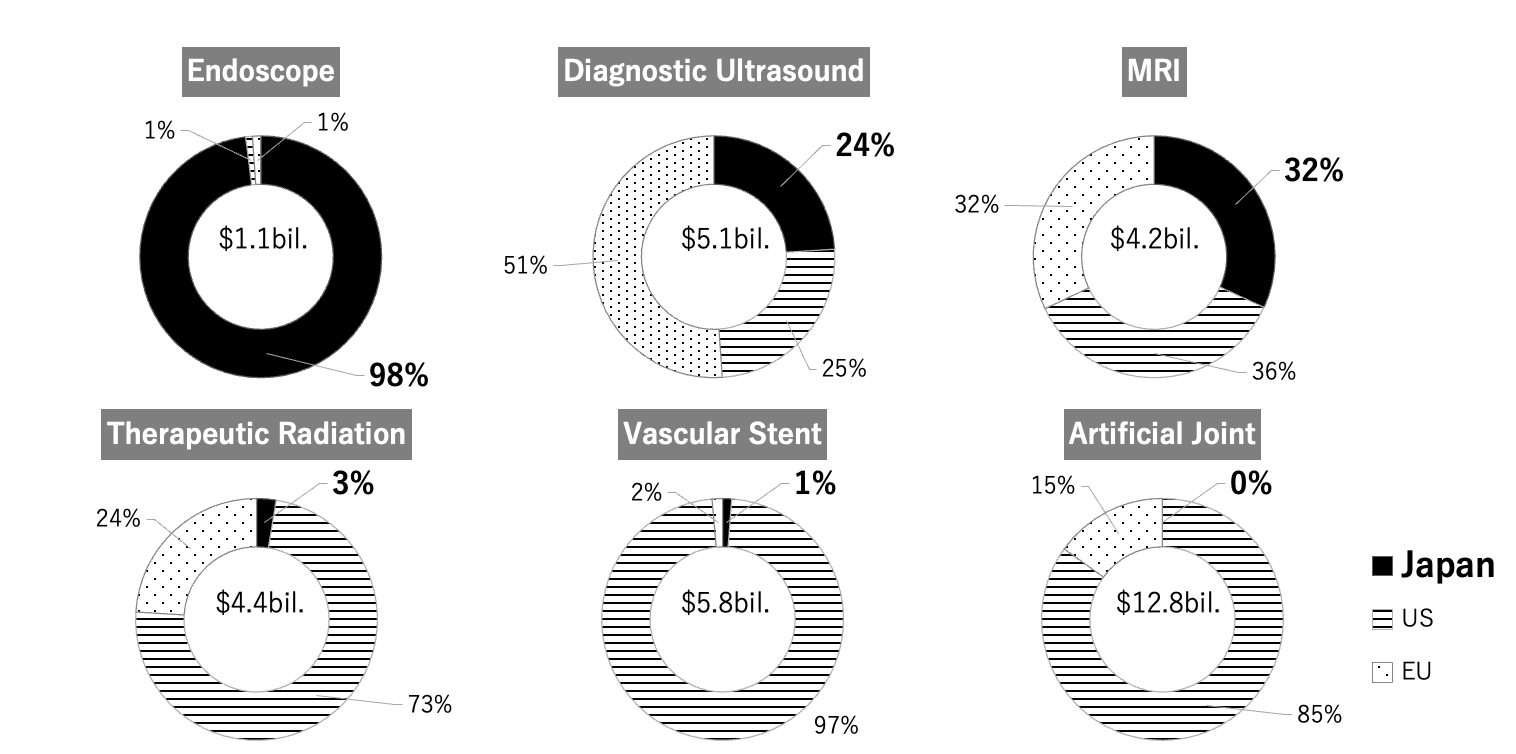

Supplement: S1 Fig — The figure shows that the Japanese companies dominate the global market share of the endoscopic market. (DOCX) [file pdig.0000001.s003.docx]
