# Supplementary material for: Regulatory-approved deep learning/machine learning-based medical devices in Japan as of 2020: A systematic review
Source: PLOS Digit Health. 2022 Jan 18;1(1):e0000001. doi: 10.1371/journal.pdig.0000001 (PMC9931274; doi:10.1371/journal.pdig.0000001)
Supplement: Attachment — Submitted filename: Point_by_point_response_20210909.docx [file pdig.0000001.s004.docx]

Reviewer 1’s comment

This paper is a systematic review article that summarizes ML/DL in medical devices in Japan. Considering Japan's position in ICH, the summary of regulation-applied M/DL-based software as a medical device in Japan has a significant meaning in digital health.

Response to reviewer

Thank you very much for your encouraging comments. We believe as you do that the current result has a significant meaning in digital health. We have revised our manuscript in accordance with your suggestions/comments.

Changes in the manuscript

-

Reviewer 1’s comment

1. It points out errors in the methodology. The authors say that if the marketing authorization holder refuses to provide information or does not respond to an email, this classification has an error. It means just the company did not provide the data from the authors' requests.

Even if the company did not provide information, the authors should add more information about these technologies by utilizing public data registered in the regulation authorization such as medical specialty, country, image type, etc. can be obtained from public data.

If this information is not added, this study lists only 11 technologies obtained from six companies and cannot be considered a review of Japan's ML/DL-based SaMD. Therefore, it is very limited information, and it has low research value.

Response to reviewer

Thank you so much for your insightful suggestion.

Although this is a common method for systematic review that is usually utilized in Cochran review etc., it can be a flaw if many companies refused to provide information. However, in reality, as we had shown (Line 174 in the original manuscript), the marketing authorization holders responded to our emails and provided information for 28 of the 30 SaMD; hence, we believe this method did not cause an error. Because Figure 3 might have been misleading, we have clarified the above explanation in our revised **Figure 3** (right).

In accordance with your suggestion, we have clarified the two SaMD for which the marketing authorization holder did not respond to our email, and summarized their medical specialty, country, image type, etc. The information is based on the publicly available data obtained from the internet. This information is provided as **Supplementary Table 1** in our revised manuscript.

Changes in the manuscript

1. We received a reply for 28 products and, among them, two were confirmed as ML/DL-based. The details of these two devices for which we did not receive a reply are presented in **Supplementary Table 1**. Consequently, 11 ML/DL-based SaMD were finally identified.

(Page12, Lines 175-176)

2. We have revised **Figure 3** to clarify the number of replies we received (or not) from the marketing authorization holders.
3. We have added **Supplementary Table 1.**

Reviewer 1’s comment

2. If this study is about regulation-approximated ML/DL-based SaMD in Japan, we would like a brief description of the approval process of SaMD in Japan PMDA and similarities/differences in FDA.

Response to reviewer

Thank you for your advice. We have briefly summarized the approval process of SaMD in Japan PMDA below. Because we are not familiar with the approval process of SaMD by the FDA, we did not refer to their similarities/differences, to avoid providing incorrect information.

The manuscript has been revised as follows below. Additionally, the summarized information is provided as **Supplementary Note 1**. Thank you for your informative suggestion.

Changes in the manuscript

Nevertheless, as the Japanese Ocular Imaging registry was established as a platform for industry-university collaboration, similar to the Intelligent Research in Sight registry of the American Academy of Ophthalmology, the number of ML/DL-based ophthalmological device will hopefully increase. Building a platform for AI utilization is an important issue in every country, and Japan is moving toward the realization. In addition, to support a rapid implementation of AI-based SaMD, the Ministry of Health Labor and Welfare and PMDA is taking a strategic approach to promote early approval of SaMD. This package is named the Digital Transformation Action Strategies in Healthcare (DASH) for SaMD. More details of DASH for SaMD are provided in **Supplementary Note 1**.

(Page 20, Lines 285–289)

*Digital Transformation Action Strategies in Healthcare for software as a medical device*

The Ministry of Health Labor and Welfare and PMDA are taking a strategic approach to promoting early approval of SaMD. This package is named the Digital Transformation Action Strategies in Healthcare (DASH) for SaMD. This package of strategies incorporates the following strategies: 1) promote the identification of innovative seeds of SaMD in the early phase to clarify the approval criteria in advance, 2) establish a unified consultation desk that provides the appropriate contact according to the contents of the consultation, 3) update the approval procedures to address the particularities of SaMD, and 4) strengthen the organizational structure involved in the review process of SaMD.

In particular, a new approval system called “Improvement Design within Approval for Timely Evaluation and Notice” (IDATEN) has been introduced to simplify the approval process for partial change. “IDATEN” is named after Mahayana bodhisattva Idaten, who is the god of speed in Japan. PMDA’s official announcement (24th May, 2021) explains IDATEN as follows: “In light of the nature of medical devices that undergo ongoing modifications and improvements during their post-market lifecycles, and AI-based programs and software whose performance is constantly changing and improving, change plans will be confirmed during the approval review process so that partial amendments to approvals can be made promptly within the scope of such plans during the devices’ post-market lifecycles.” (https://www.pmda.go.jp/english/about-pmda/0006.pdf, accessed on 3rd, September, 2021).

(**Supplementary Note 1**)

Reviewer 1’s comment

3. Further investigation is needed to determine how much ML/DL-based SaMD is currently being used within the Japanese healthcare market. Discussion said that the application of relatively more ML/DL-based SaMD to the endoscope field than the U.S. reflects the Japanese medical market. However, it is also necessary to provide evidence for actual sales and use.

Response to reviewer

We apologize for the confusion. In the discussion section, we did not refer to the sales and use of endoscopy-related ML/DL-based SaMD but to those of endoscopic devices (hardware) here.

Considering your suggestion, we have provided the evidence for actual sales and use of endoscopic devices (hardware).

This figure shows the global market share in each medical devices field. The original data are provided by the Japan Ministry of Economy, Trade, and Industry since February 2021 (available at <https://www.med-device.jp/repository/meti-seisaku-202002.pdf>, accessed on 20^th^ August 2021). Because the original data were provided in Japanese, we translated them into English. Japanese Yen was converted to US dollars based on an exchange rate of 2014 ($1≒109.66yen). The figure shows that the Japanese companies dominate the global market share of the endoscopic market (98%). We speculate that this fact might be a reason for the relatively more application of ML/DL-based SaMD to the endoscope field in Japan than in U.S.

We have provided the Figure as **Supplementary Figure 1**. Thank you very much for your helpful advice.

Changes in the manuscript

Moreover, the high proportion of ML/DL-based SaMD related to endoscopy in Japan reflects Japan’s industrial strength in endoscopic systems (**Supplementary Figure 1**).(21)

(Page 20, Line 297)
